# A standardized approach to study human variability in isometric thermogenesis during low-intensity physical activity

**DOI:** 10.3389/fphys.2013.00155

**Published:** 2013-07-01

**Authors:** Delphine Sarafian, Jennifer L. Miles-Chan, Gayathri Yepuri, Jean-Pierre Montani, Yves Schutz, Abdul G. Dulloo

**Affiliations:** Department of Medicine/Physiology, University of FribourgFribourg, Switzerland

**Keywords:** obesity, spontaneous physical activity, NEAT, exercise, thermogenesis, energy expenditure, isometric, static

## Abstract

**Limitations of current methods:** The assessment of human variability in various compartments of daily energy expenditure (EE) under standardized conditions is well defined at rest [as basal metabolic rate (BMR) and thermic effect of feeding (TEF)], and currently under validation for assessing the energy cost of low-intensity dynamic work. However, because physical activities of daily life consist of a combination of both dynamic and isometric work, there is also a need to develop standardized tests for assessing human variability in the energy cost of low-intensity isometric work.

**Experimental objectives:** Development of an approach to study human variability in isometric thermogenesis by incorporating a protocol of intermittent leg press exercise of varying low-intensity isometric loads with measurements of EE by indirect calorimetry.

**Results:** EE was measured in the seated position with the subject at rest or while intermittently pressing both legs against a press-platform at 5 low-intensity isometric loads (+5, +10, +15, +20, and +25 kg force), each consisting of a succession of 8 cycles of press (30 s) and rest (30 s). EE, integrated over each 8-min period of the intermittent leg press exercise, was found to increase linearly across the 5 isometric loads with a correlation coefficient (r) > 0.9 for each individual. The slope of this EE-Load relationship, which provides the energy cost of this standardized isometric exercise expressed per kg force applied intermittently (30 s in every min), was found to show good repeatability when assessed in subjects who repeated the same experimental protocol on 3 separate days: its low *intra*-individual coefficient of variation (CV) of ~ 10% contrasted with its much higher *inter*-individual CV of 35%; the latter being mass-independent but partly explained by height.

**Conclusion:** This standardized approach to study isometric thermogenesis opens up a new avenue for research in EE phenotyping and metabolic predisposition to obesity.

## Introduction

The assessment of human energy expenditure (EE), under standardized conditions, has wide applications in human energy metabolism ranging from the estimation of energy requirements of population groups and individual hospitalized patients (Miles, 2006; Shephard and Aoyagi, 2012) to the elucidation of the genetic and metabolic basis of human susceptibility to obesity (Dulloo et al., 2012). In this context, standardized approaches for assessing human variability in EE measured at rest in the postabsorptive state as the basal metabolic rate (BMR) or in the postprandial state as the thermic effect of feeding (TEF) are well-defined (Schutz, 2008; Schutz and Dulloo, in press), as are measurements of the energy cost of moderate-to-high intensity exercise performed during treadmill walking/running, repetitive bench press and squat exercises or during cycling ergometry (Donovan and Brooks, 1977; Bijker et al., 2001; Robergs et al., 2007; Lazzer et al., 2011).

During the past decade, there has been considerable interest in the notion that EE associated with everyday life physical activities, often referred to as non-exercise activity thermogenesis, play an important role in the regulation of body weight (Dauncey, 1990; Levine et al., 2006; Garland et al., 2011). To study such low-intensity physical activities, however, is a challenging task as they include not only voluntary occupational and leisure activities but also subconscious spontaneous physical activity such as muscle tone and posture maintenance and fidgeting (Thompson et al., 2009; Westerterp, 2009). In addition to the development of accelerometers and activity monitors for the detection and quantification of these low-intensity activities (Wong et al., 2007; Melanson et al., 2009; Plasqui et al., 2013), methodological approaches are currently being developed and validated for assessing EE variability in response to standardized dynamic exercise at low power outputs that are energetically comparable to low-intensity physical activities of daily life (Reger et al., 2012). However, because movements during daily life comprise not only dynamic work but also isometric (static) work, and that intermittent isometric thermogenesis is an important component of EE associated with spontaneous physical activity (Dulloo et al., 2012), there is a need to develop a standardized test for assessing human variability in the energy cost of intermittent isometric exercise of low-intensity.

To this end, we report here the development and validation of an approach that consists of incorporating a standardized protocol of intermittent leg press exercise of varying low-intensity isometric loads with measurements of EE by indirect calorimetry in a comfortable seated position. The specific objective of the study reported here was to test the feasibility and repeatability of this approach for assessing the energy cost of isometric thermogenesis in response to such intermittent low-intensity isometric work.

## Materials and methods

### Criteria for method development

In the development of an appropriate methodological approach to study human variability in isometric thermogenesis pertaining to the field of nutrition and metabolism, several criteria were established. *First*, the standardized isometric exercise test should be feasible for incorporation with measurements of EE using indirect calorimetry by the ventilated-hood (canopy) system. *Second*, the isometric loads should be low in intensity and intermittent so as to mimic real life, with each isometric contraction alternating with a period of rest of similar duration, such that each cycle of isometric load and rest would lead to increases in EE that would be in the range of some of the low-level physical activities during everyday life, i.e., within 2-fold relative to resting levels. *Third*, the intermittent isometric loads should be low enough in intensity and short enough in duration so that min-by-min blood pressure is unlikely to increase more than marginally even at the highest intermittent isometric load. *Fourth*, the energy cost of the isometric exercise should be assessed by regression analysis of EE *vs* kg force loads applied, so as to enable the calculation of the “delta energy cost” of the exercise test (i.e., energy cost per kg force applied intermittently) by analogy to the calculation of delta mechanical efficiency for dynamic exercise.

### Standardization of posture and isometric loads

**Figure 1:**
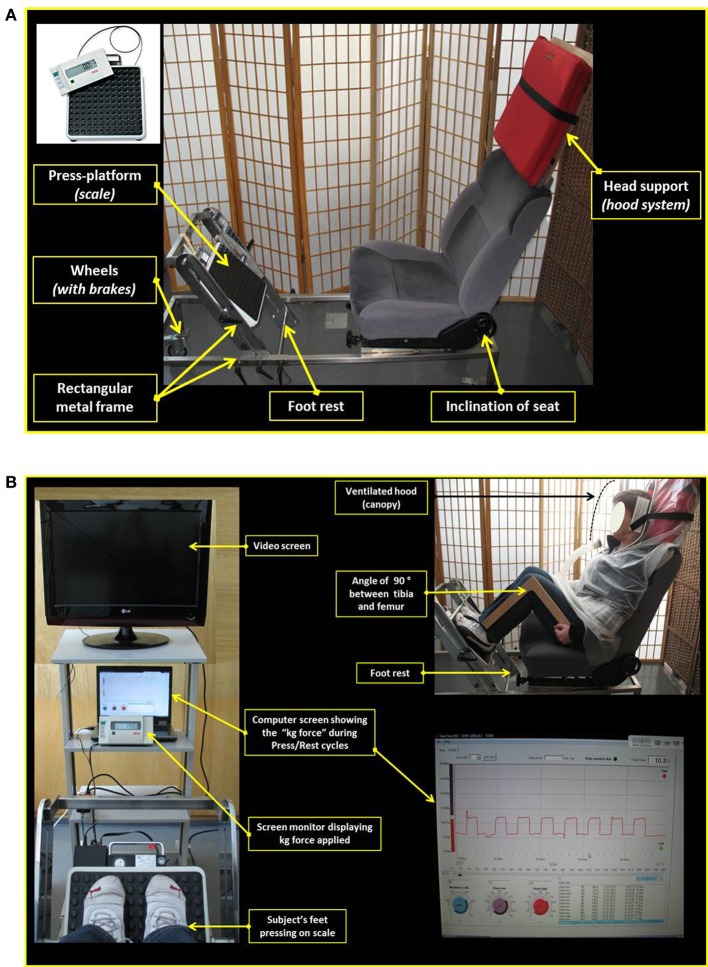
**(A)** Picture showing the adapted car seat and the press-platform used for the standardized isometric exercise test (See text for details). **(B)** Standardized posture during isometric exercise (See text for details).

EE was measured by ventilated-hood indirect calorimetry (Deltatrac II, Datex-Ohmeda, Helsinki) in a comfortable seated position in an ergonomic and adjustable car seat which was mounted on a rectangular metal frame on wheels with strong brakes (Figure 1A). In order to apply the “hood” component of the indirect calorimetry system in the seated position, the top part of the seat's back-support was modified so as to incorporate a head-support (50 cm long × 50 cm wide × 1.5 cm thick) made of a wooden base covered with a sponge-filled cushion; the angle of inclination of the seat's back support was adjusted between 110 and 120°. The standardized posture of the subject at rest during baseline measurements was to sit in the car seat with the feet on the foot rest or along the sides of the metal frame. Isometric work was performed by pressing both legs simultaneously against a press-platform which consisted of an in-built metal frame carrying a weighing scale (Seca 862, Hamburg, Germany) that could tilt around its horizontal axis. The position of the press-platform, which could slide horizontally along the metal frame, is adjusted for each subject such that when the subject's feet are at rest and flat on the press-platform, the angle between subject's thigh (femur) and lower leg (tibia) is a right angle (90°). Under these conditions, the value obtained (without any active leg press) corresponded to the kg force exerted by the weight of the legs on the press-platform. This “passive leg load” was found to be highly reproducible both within and between days in a given subject, and to vary between 10 and 20 kg among the subjects participating in the experiment reported here. For each subject, this *passive* leg load is taken as a reference value which is then used to define 5 different *active* press load levels (i.e., 5 different isometric loads), namely +5, +10, +15, +20, and +25 kg force, and referred to as isometric load L1, L2, L3, L4, and L5, respectively.

### Data acquisition and visual display

The output of the weighing scale is relayed, through a digital monitor screen (Seca 862, Hamburg, Germany), to a laptop computer containing a custom-built software for data acquisition and continuous second-by-second screen display of the real-time kg force exerted on the press-platform (Figure 1B). Both the digital monitor screen and the computer laptop/screen are placed on a rack which is positioned approximately two meters in front of the subject. Prior to each exercise test, the set isometric load (kg force) that the subject should exert on the press-platform is introduced in the computer. During the leg press exercise, the actual isometric load exerted on the press-platform is continuously read by the computer software, and any deviation of 1 kg above or below the set isometric load triggers an electronic “beep” alarm which alerts the subject to maintain the leg press within ± 1 kg of the set load. The onset and termination of each press/rest cycle is signaled by the investigator.

### Design of isometric exercise

**Figure 2:**
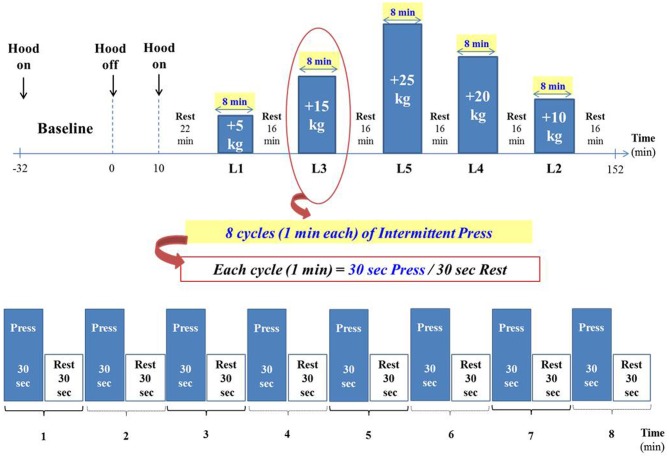
**Experimental design (See text for details)**. The duration of the entire exercise test is approximately 2.5 h, after 30 min of baseline measurements.

Preliminary experiments were undertaken to establish the duration and number of press/rest cycles in line with the criteria set above. These criteria were met by performing a succession of 8 cycles of press/rest for each isometric load level, with the press (contraction) and rest (relaxation) periods lasting 30 s each. During the 30 s of rest within each cycle, the legs are kept “passively” on the press-platform (i.e. without exerting any active press). After each set of exercise bouts (i.e., after 8 press/rest cycles at a given isometric load level), the subject remains seated at rest for another 16 min, with the feet along the sides of the metal frame (as during the baseline period). The experimental design for a typical experimental run is presented schematically in Figure 2; the sequence of the 5 press loads applied during each experimental run for each subject is as follows: L1, L3, L5, L4, and L2.

### Data analysis

**Figure 3:**
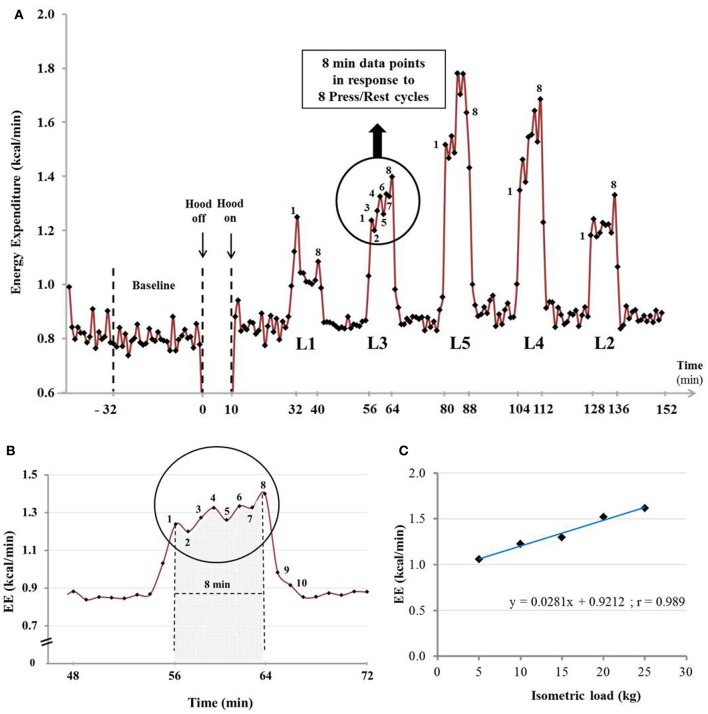
**(A)** Example of the EE (kcal/min) profile in a young woman at rest (Baseline) and in response to the five isometric active loads (L1, L2, L3, L4, L5) recorded minute by minute by indirect calorimetry. The isometric (active) loads are: L1 = +5 kg, L2 = +10 kg, L3 = +15 kg, L4 = +20 kg and L5 = +25 kg. **(B)** Illustration of the computational method used for data analysis. The EE profile during isometric exercise at L3 (as shown above) is magnified. **(C)** Linear regression analysis on mean EE (kcal/min) data as a function of five isometric active loads (+5, +10, +15, +20, +25 kg). Note that this subject is not included in those who participated in the subsequent (repeatability) experiment.

An example of the EE profile in response to the five press loads in a young woman is shown as min-by-min values (kcal/min) in Figure 3A. At a given press load level, each EE data point is an integrated 1 min EE value; this corresponds to EE for one press/rest cycle, with each cycle consisting of 30 s of “press” and 30 s of “rest.” Within the black circle which emphasizes the 8 min-by-min data points of EE in response to the 8 press/rest cycles at press load level 3 (L3), and amplified in Figure 3B, the data point labeled “1” corresponds to the EE in response to the 1st press/rest cycle, and so on, with the data point labeled “8” corresponding to the last minute EE data in response to the 8th press/rest cycle. The overall data of EE in response to each press load level—comprising the 8 press/rest cycles—is analyzed as the integrated mean EE of the 8 min EE data points corresponding to the 8 press/rest cycles; this is analogous to the method for calculating the energy cost (and delta efficiency) of dynamic work. For each run, a plot of EE *vs* isometric loads yields a strong linear relationship, with the correlation coefficient *r* > 0.9, as shown in Figure 3C. The slope of this linear regression therefore provides the energy cost of performing the standardized isometric exercise per kg force applied half of the time (i.e. per kg force t_1/2_); this is referred to as the “delta energy cost” of the exercise, and can be calculated from regression lines that either exclude or include the baseline EE (BEE) value (i.e., EE at 0 isometric load), and expressed as kcal.min^−1^ / kg force t_1/2_.

### Cardiovascular monitoring

Non-invasive cardiovascular recordings were performed using a Task Force Monitor (CNSystems, Medizintechnik, Graz, Austria) as described previously by Brown et al. (2008). Cardiac intervals (and their reciprocal, heart rate) were recorded by electrocardiography. Four ECG electrodes were placed on the subject's torso and thoracic impedance was recorded using three band electrodes, one placed on the back of the neck and two parallel electrodes placed on the lateral sides of the thorax at the level of the xiphoid process. Electrocardiogram and impedance cardiogram cables were then connected. Cardiac output and stroke volume were derived on a beat-to-beat basis from the impedance cardiogram (Kubicek et al., 1996). Continuous blood pressure was recorded during the baseline period and during the highest isometric load exercise (L5) from the index and middle fingers of the right hand alternatively using the vascular unloading technique, and calibrated to oscillometric brachial blood pressure measurements on the contralateral arm.

### Subjects and repeatability experiment

To assess the intra-individual variability in the energy cost of isometric exercise, the standardized leg press exercise at various isometric loads was conducted in 9 young non-obese adults (4 men and 5 women; age range of 21–29 years), with the test repeated in the same subject on 3 different days and with at least a two days interval between any 2 test days. All measurements were performed in the morning after an overnight fast, and after the determination of baseline EE at rest (BEE) over at least 30 min. None of the subjects were athletes or engaged in regular sports activities on daily or weekly basis. They all, however, considered themselves as being moderately active and fit, spending between 30–60 min walking during their daily activities. All subjects maintained a relatively stable body weight (within ± 1 kg) during the 3 months preceding the study. Exclusion criteria were as follows: regular smokers, claustrophobic subjects, pregnant or breastfeeding women, subjects with acute infections, chronic inflammatory disease or taking medication which could interfere with metabolic rate. Participants were asked to avoid intense physical activity and to abstain from caffeine-containing foods and beverages for 24 h before the test. Subjects were also required to eat their dinner before 20 h on the eve of the test, in order to comply with 12 h of fasting. All women were tested in the follicular phase of their menstrual cycle. To minimize the effect of physical activity on the morning of the test day, participants were requested to use motorized transport instead of walking or cycling to reach the laboratory. Before enrollment in the study, the participants came to the laboratory for an interview where they completed a questionnaire about their lifestyle and medical history; anthropometry measurements (weight and height) were made using a mechanical scale with an integrated stadiometer (Seca model 709, Hamburg, Germany). On this occasion, the study objectives and procedures before and during the experiments were explained, and a written informed consent was obtained for all subjects. All procedures complied with the Declaration of Helsinki and received local ethics committee approval.

## Results

### EE-load relationship

**Figure 4:**
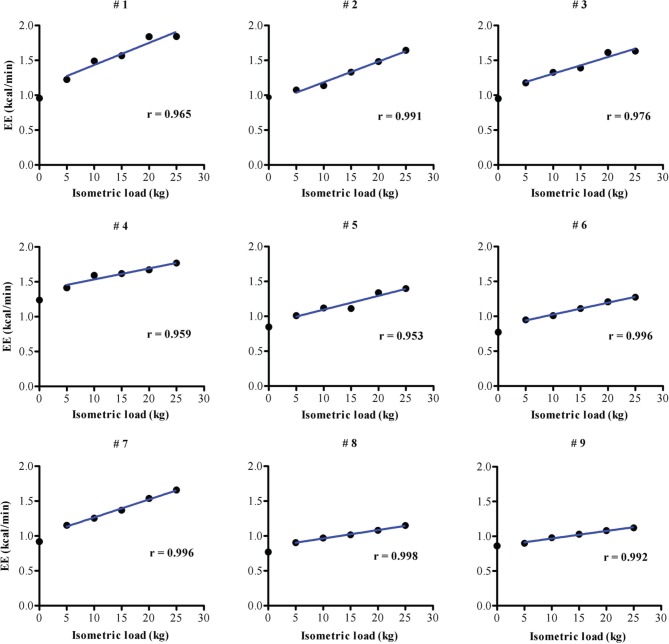
**Linear regression of EE (kcal/min) as a function of the five isometric active loads (+5, +10, +15, +20, +25 kg)**. The coefficient of regression (*r*) is shown for each subject. # denotes subject number.

**Figure 5:**
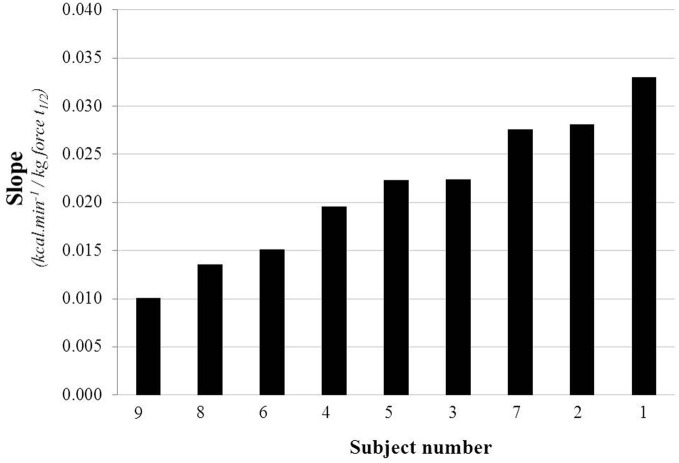
**Distribution of the slope values (3d-mean) assessed by linear regression on three separate days among the nine subjects**.

The EE vs. isometric load regression line for each of the nine subjects on the first of their three separate test measurements is shown in Figure 4. Overall, EE is found to increase above baseline by 11–93% across the various loads L1–L5 (+5 to +25 kg force), with a strong linear EE-Load relation (*r* > 0.95) for each subject. The distribution of the slope values, presented in ascending order in Figure 5, indicates a large inter-individual variability in the energy cost of performing this standardized exercise (>3-fold difference in slope values (range: 0.01–0.033). The value of the y-intercept (EE at zero isometric load) is found to be significantly higher (*p* < 0.01) than the measured BEE value by 0.063 kcal/min: an increase of EE above baseline of 6.7% on average, with 8 of 9 subjects showing y-intercept higher than BEE in the range of 3–12%. No significant differences were found between men and women in the values of y-intercept minus BEE, and in the slope values.

### Intra-individual variability in EE

**Figure 6:**
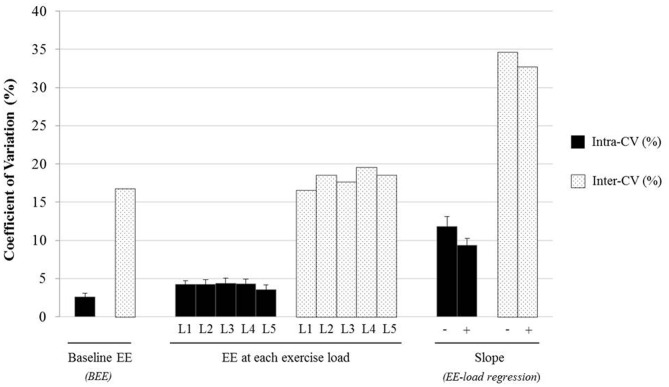
**Intra- and inter-individual coefficient of variation (CV%) of EE at rest and during isometric exercise in 9 young adults**. The CV (%) was determined according to EE measured at baseline (BEE), EE at each active isometric load (L1, L2, L3, L4, L5) and for EE-Load regression slope measured on three separate occasions (3d-mean EE) with BEE excluded (−) or included (+) in the regression analysis. Values are Mean ± SEM for intra-CV and mean values for inter-CV.

The data on coefficient of variation (CV%), based on three repeated measures on the three separate days for BEE and EE at each isometric load, as mean values for the nine subjects, are presented in Figure 6. As expected, across these 3 days, the intra-individual CV (intra-CV) of BEE, like that often reported for BMR (Adriaens et al., 2003), is within 5% with a mean intra-CV of 2.6% and range of 0.5–5%. At each press load (L1–L5), the mean intra-CV value for EE is also found to be <5%, with the individual intra-CV in the range of 1–8% across the various press loads. There are no significant differences in the mean intra-CV for EE at each exercise load, with values for L1, L2, L3, L4, and L5 being within the narrow range of 3.6–4.4%. Furthermore, at any press load, the mean intra-CV (~4%) is several times (4–5 folds) lower than the inter-individual CV (inter-CV) values which range between 16.6 and 19.6%.

### Intra-individual variability in slope of EE-load relationship

Comparison of the delta energy cost of the standardized exercise, assessed from the slope of the regression between EE and isometric loads, indicates that the large inter-individual variability among the nine subjects (mean inter-CV ~ 35%) is 3 fold greater than the mean intra-CV value of 11.8% (range 6.4–17%) (Figure 6, right-hand columns). The inclusion of BEE (i.e., the measured EE at zero isometric load) in the regression analysis yielded slightly but significantly higher slope values (0.023 vs. 0.021, *p* < 0.01), as well as slightly but significantly lower mean intra-CV (9.3 vs. 11.8%, *p* < 0.05), with the mean inter-CV being 3.5 fold greater than the mean intra-CV, as shown in Figure 6 (right-hand bars).

### Training/habituation and load sequence effects

**Figure 7:**
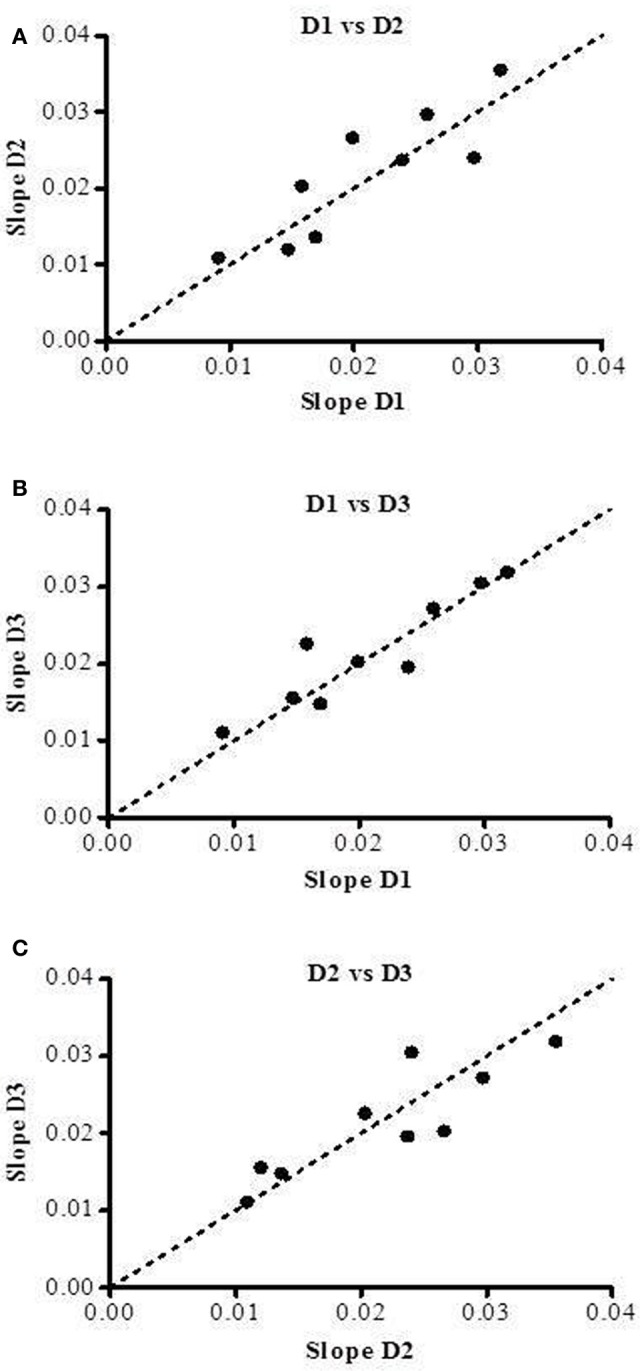
**Slope values obtained on the three separate days of testing (D1, D2, and D3) in nine subjects**. Slope values of Day 2 and Day 3 are plotted against slope values of Day 1 (panels **A,B**) and slope values of Day 3 are plotted against slope values of Day 2 (panel **C**). The dotted line corresponds to the line of identity.

To test whether there is a systematic trend of changes in the slope values across the three measurement days, the ratio of the slopes of the EE-Load relationship were examined as follows: “Day 2 vs. Day 1,” “Day 3 vs. Day 1,” and “Day 3 vs. Day 2,” and presented in Figure 7. The results indicate no systematic directional change in the slope values across the measurement days, thereby suggesting that there is no systematic training or habituation effect in the assessment of the energy cost of the exercise. Furthermore, the findings that the performance of the L4 and L2 loads after the highest load L5 (i.e., order sequence: L1, L3, L5, L4, L2) has no impact on the linear relationship between EE and isometric loads L1–L5 suggest that there are no time-dependent effects and no hysteresis in the observations.

### Overall findings in EE vs. load relationship

This experiment above designed primarily to investigate the within-subject variability in the delta energy cost of the standardized isometric exercise suggests a much lower intra-individual variability compared to the inter-individual variability (intra-CV of ~10% vs. inter-CV of 35%), and that the intra-CV in the delta energy cost of the standardized isometric exercise is not significantly different in men and women.

### Anthropometric correlates with EE in response to press loads

Analysis of anthropometric predictors of the large inter-individual variability in the energy cost of the isometric exercise at each load (total EE − BEE) as well as in the slope of the EE-Load regression show no correlation with body weight or with lower weight exponents (Table 1), as well as with the passive load (i.e., the kg force exerted by positioning the legs on the press-platform but without actively pressing); there was however a significant (or close to significant) association with height. This contrasts with baseline EE, which as expected, is found to be strongly correlated with both body weight and height.

**Table 1:** **Pearson's correlation analysis of baseline energy expenditure (EE) or BEE, activity energy expenditure (change in EE above baseline) and slope of EE-load regression vs anthropometry (body weight, lower body weight exponents, and height), as well as vs. the passive load (the kg force exerted by the weight of the legs on the press-platform)**.

		**Baseline EE (BEE)**	**Slope (EE-load regression)**	**Change in EE above baseline**				
				**+5 kg**	**+10 kg**	**+15 kg**	**+20 kg**	**+25 kg**
**BODY WEIGHT (BW)**								
BW^1^	*r*	0.84	0.17	0.14	−0.03	0.16	0.02	0.18
	*p*	<0.01	NS	NS	NS	NS	NS	NS
BW^0.7^	*r*	0.83	0.18	0.13	−0.03	0.16	0.02	0.19
	*p*	<0.01	NS	NS	NS	NS	NS	NS
BW^0.5^	*r*	0.82	0.18	0.12	−0.03	0.16	0.02	0.19
	*p*	<0.01	NS	NS	NS	NS	NS	NS
BW^0.3^	*r*	0.82	0.19	0.12	−0.03	0.15	0.02	0.19
	*p*	<0.01	NS	NS	NS	NS	NS	NS
BW^0.1^	*r*	0.81	0.19	0.11	−0.03	0.15	0.03	0.20
	*p*	<0.01	NS	NS	NS	NS	NS	NS
Passive load	*r*	0.60	−0.03	−0.04	−0.21	−0.05	−0.18	−0.02
	*p*	=0.08	NS	NS	NS	NS	NS	NS
Height	*r*	0.79	0.70	0.71	0.54	0.77	0.63	0.76
	*p*	<0.01	<0.05	<0.05	=0.14	<0.05	=0.07	<0.05

*NS, not statistically significant (p > 0.05); the p-values are also indicated for r-values close to statistical significance (p-value between 0.05 and 0.15)*.

### Cardiovascular responses

**Figure 8:**
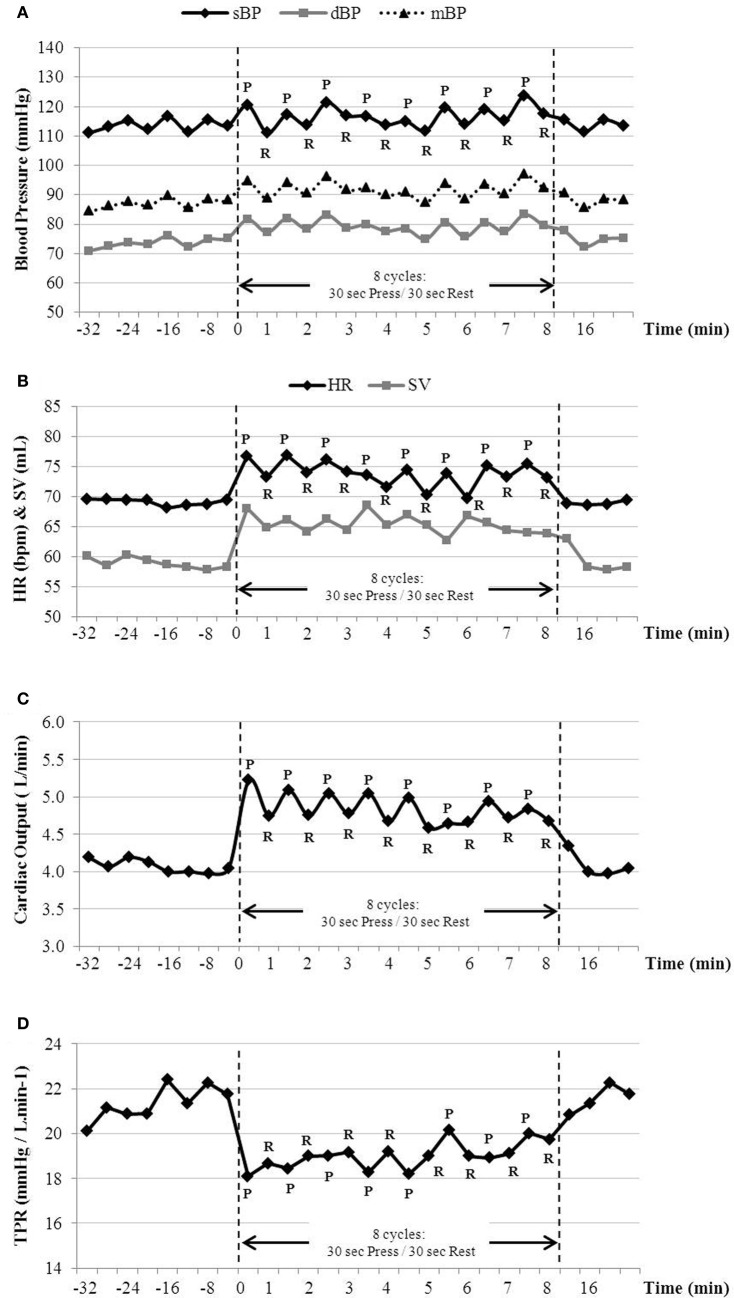
**Cardiovascular parameters in response to the highest isometric load (*L*5 = +25 kg) in one subject (#8)**. For the 8 press/rest cycles performed at the highest load, **(A)** blood pressure (BP), **(B)** heart rate (HR), stroke volume (SV), **(C)** cardiac output and **(D)** total peripheral resistance (TPR) were averaged as 30 s during leg press (P) and rest (R). Each data point before and after pressing at the highest load represents rest periods and were processed as 4 min means.

**Figure 9:**
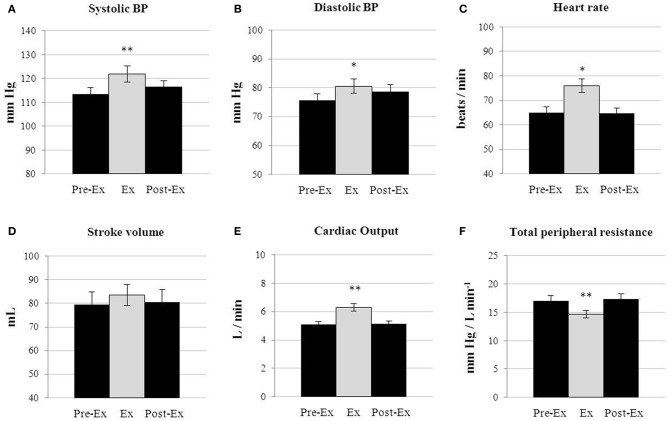
**Effect of the intermittent isometric exercise on hemodynamics**. Cardiovascular parameters (blood pressure, heart rate, stroke volume, cardiac output and total peripheral resistance) in response to the highest press load (+25 kg force) in subjects (*n* = 7), and compared to the rest periods before (Pre-Ex Rest) and after (Post-Ex Rest) the highest press load (Ex). Statistical significance of differences is indicated with the symbol * denoting statistical significance as assessed by ANOVA repeated measures; ^*^p < 0.05, ^**^p < 0.01.

To validate that this approach to study the energy cost of isometric work does not lead to overt increases in total peripheral resistance (TPR) and blood pressure, as would be expected for isometric loads that are intermittent, of relatively short duration (30 s) and low in intensity (<30 kg force applied with the legs, and <2-fold increase in EE above baseline), a comprehensive analysis of the cardiovascular response was monitored in subjects (*n* = 7) during baseline period at rest, during their highest press load, i.e., during the 8 press/rest cycles at press load L5, and during another 16 min after the press load exercise. A detailed 30 s. by 30 s. cardiovascular response to the intermittent isometric exercise for one individual (subject no. 8) is shown in Figure 8, and the results for all subjects as means of 16 min baseline followed by 8 min of intermittent exercise at highest press load (L5), and followed for another 16 min post-exercise are presented in Figure 9. In all these subjects, there were significant but small increases (<8 mm Hg) in systolic and diastolic blood pressure (Figures 9A,B) during the 8-min of intermittent exercise. Cardiac output was found to be significantly higher (+23%) and TPR to be significantly lower (−14%) during the exercise than at rest (panels E and F, respectively). The increase in cardiac output was characterized by an increase in both heart rate (panel C) and stroke volume (panel D).

### RQ-load relationship

**Figure 10:**
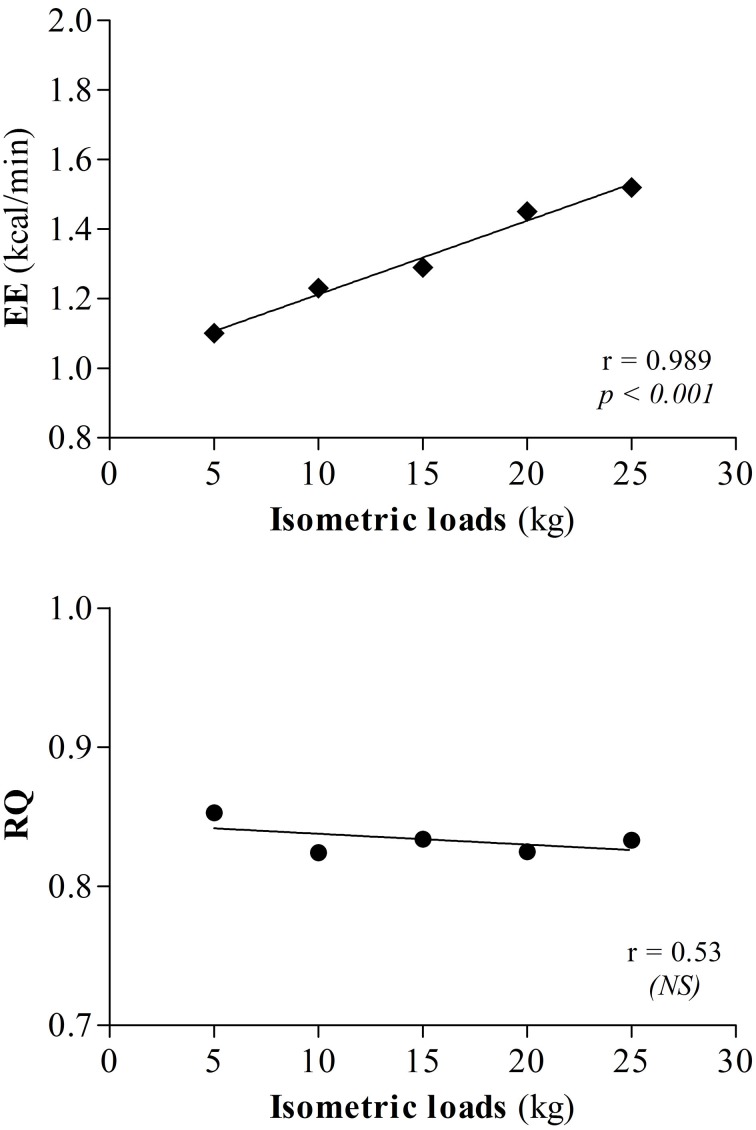
**Linear regression of EE (kcal/min) or respiratory quotient (RQ) as a function of the five isometric active loads (+5, +10, +15, +20, +25 kg)**.

Unlike for the EE-load relationship showing a robust linear regression of EE vs. isomertric loads applied, no significant relationship is found between RQ and the isometric loads (Figure 10).

## Discussion

The primary objective of this study was to extend the capacity for metabolic and EE phenotyping beyond those conducted in the resting state (BMR, TEF) or during dynamic exercise, by developing a standardized approach to study human variability in isometric thermogenesis in response to intermittent low-intensity isometric leg press exercise. In this methodological development, several considerations and criteria were taken into account in line with the long-term objective of applying this approach for investigations in the area of human nutrition and weight regulation. These are discussed below, first in relation to the practicality and feasibility of conducting the exercise protocol, and subsequently in relation to analytical issues in determining the energy cost of the isometric exercise.

### Sitting posture and seated exercise

As a first step in the development of the standardized exercise, it was important that the study was conducted with minimum discomfort for the subjects, while at the same time ensuring that the exercise test at different isometric loads would lead to increases in EE in a range compatible with low-intensity physical activity of everyday life. To this end, the subjects were seated in an ergonomic and adjustable car seat that was modified for enabling continuous assessment of EE with the subject at rest or while exerting intermittent leg press—an isometric exercise that would involve a large skeletal muscle mass. The seated position is also convenient for the subject to execute the isometric exercise as it allows easy reading of the visual feedback (Seca monitor) to check if the amount of force pressed on scale is close (±1 kg) to the pre-defined target value. Furthermore, in order to prevent boredom during the various rest periods between exercise bouts, the seated position provides a suitable posture for watching a documentary/film on a video screen placed on a rack in front of the subject. Overall, the entire protocol, which involves continuous monitoring of EE by indirect calorimetry during the various periods of rest and intermittent leg press exercise, was found to be simple and easy to perform by the subjects, and feasible to be co-ordinated by the investigator.

### Ventilated-hood calorimetry

Breath-to-breath gas analysis by an indirect calorimetry system with the subject wearing an appropriate facemask or mouth-piece/nose-clip is generally applied to monitor EE during exercise. Although it is not feasible to monitor high rates of EE of several METs by the ventilated-hood technique—due to non-adjustable air flow rate limitations and CO_2_ build-up—this system can nonetheless be used to assess much more modest increases in EE such as during our study involving low-intensity and intermittent leg press exercise where EE is below 2 METs even at the highest press load. Indeed, in our study EE was found to increase linearly above baseline by 17–62% on average across the 5 loads levels, with the lowest individual value at L1 (+5 kg) and highest individual value at L5 (+25 kg) corresponding to increases in the range of 11–93%. This range is comparable to increases in EE above supine levels observed during standing motionless (+13%) (Levine et al., 2000), sitting while typing (+50%) (Ainsworth et al., 2011) and during standing while fidgeting (Levine et al., 2000) or during cycling against zero watt (no-load cycling) (+70 to 90%) (Reger et al., 2012), and is clearly below the 3–4 fold increase in EE observed during walking on a treadmill at 3–5 km/h (Levine et al., 2000). The incorporation of a head-support to the back of the seat allowed EE to be measured by the ventilated-hood indirect calorimetry system in the seated position. The fact that the exercise test involves only the lower part of the body (i.e., both legs) makes the measurement easy to perform with the components of the ventilated-hood system (hood and plastic veil) covering upper body while sitting at ease. The advantage of the ventilated-hood system is that it allows the measurement of gas exchange from an individual's “natural” breathing, which is often reported to be more comfortable than when using a facemask or mouth-piece with nose-clip; these tending to be more stressful, leading to overestimation of EE. Indeed, EE at rest in subjects wearing a mouthpiece/nose-clip system or face-mask system have been found to be 6-8% higher than when measured by the ventilated hood system (Forse, 1993; Roffey et al., 2006). Moreover, the use of the ventilated-hood system is more suitable for studies that are of long duration (several hours) as in our test where several periods of rest are needed after each isometric load level.

### Bypassing reduced muscle blood flow limitations

An assumption of our study is that the intermittent low-intensity contractions of 1 min cycles of 30 s press/30 s rest would not result in any sustained reduction in blood flow and hence would not induce increased lactate formation as has been demonstrated when static contraction is sustained for several minutes or until exhaustion (Cerretelli et al., 1976; Gorostiaga et al., 2010). Whilst we did not measure muscle lactate formation or blood lactate concentrations, our findings showed that the min-by-min TPR was reduced (by 14% on average) during the highest isometric load applied, suggesting that min-by-min blood flow actually increased during intermittent isometric exercise under conditions of our study. Indeed, the significant reduction in TPR counteracted much of the expected large increase in blood pressure classically seen in sustained isometric exercise (Friedman et al., 1992) leading to only small increases (<8 mm Hg) in blood pressure even at the highest intermittent isometric load. Since isometric contractions were alternated with equal time periods of rest in our standardized test, the diminution in TPR observed in our results can be explained by similar phenomenon to that occurring in dynamic exercise where TPR decreases due to vasodilation of the arterioles, allowing more blood and oxygen into the working muscle. Furthermore, compared to an intermittent static exercise performed with the arms (handgrip tests), the larger muscle mass recruited in our task (both legs) would induce a greater vasodilatation and might explain the observed decrease in TPR.

### Choice of isometric load standardization

By comparison with standardized dynamic exercise (i.e., using a cycle ergometer or treadmill) where the intensity of physical activity is generally expressed as a percentage of VO_2_ max and the power output measured in watts, most studies related to isometric exercise have defined the intensity of workloads as a percentage of maximal voluntary contraction (MVC). However, depending on both physical and mental status (co-operation, motivation) of the subject and the type/nature of the isometric exercise, the determination of the MVC during isometric tests can be highly variable within the same individual; i.e., it has a poor reproducibility. In preliminary experiments in our laboratory on four healthy young men aimed at assessing within-subject variability in maximum leg press isometric contraction performed in our car seat set-up, we found CV% as high as 40–70% for MVC (data not shown); these latter findings led us to abandon the use of percentage MVC in the standardization of the exercise load in our study. The use of a percentage of body weight instead of a percentage of MVC has been suggested to be a more appropriate measure of load level (Kondraske et al., 1987), but as our study was conducted in the seated position, and hence the body weight mostly supported by the seat, it seems more relevant that the isometric load to be exerted on the press-platform was determined as a function of weight of the subject's leg on the press-platform. The “passive” leg load, which ranged between 10 and 20 kg in our subjects, was used to define the 5 press load levels during our exercise test by adding to each subject's passive leg load, the five different active loads (+5, +10, +15, +20, +25 kg). The sequence of isometric loads was fixed for all participants and consisted of a mixture of ascending and descending intensity force level during the exercise test in order to avoid any systematic ordering effects. Under these conditions, the strong linear relationship between EE and the isometric loads applied intermittently (*r* > 0.9 for all subjects) provides an appropriate analytical approach for determining the energy cost of the standardized exercise by linear regression and to phenotype individuals for thermogenesis in response to intermittent isometric work, and hence in variability in isometric thermogenesis.

### Linearity of EE-load relationship

Because this analytical approach—based upon EE-Load linear regression—is analogous to the calculation of delta efficiency in dynamic exercise, it is referred to here as the “delta energy cost” of the isometric exercise. It is to be noted that amidst decade old debates regarding the ideal efficiency calculation for dynamic exercise, the calculation of delta efficiency is believed to most closely approximate muscle efficiency (Gaesser and Brooks, 1975; Poole and Henson, 1988; Ettema and Loras, 2009). Furthermore, as recently emphasized by Reger et al. (2012) in referring to the delta efficiency for dynamic exercise, “*the slope may be the best indicator of exercise efficiency since the slope reflects the metabolic cost of biological processes that increase as power outputs increase*.” In the field of Physics, however, the term “efficiency” does not apply to isometric work since the definition of physical work (Joule) implies a force (Newton) and displacement (meter). But in isometric or static work, there is no muscle shortening (displacement)—as in our task where both legs of the participant are pushing against a fixed, stationary platform without moving it through a distance; in other words, according to the physics definition, no external work is performed on the environment. Consequently, in our study focused on measurements of EE during isometric exercise, the strong EE-Load linear regression observed allows the calculation of the delta energy cost of the standardized isometric exercise (expressed per kg force applied intermittently).

### Reliability in assessing the energy cost of isometric exercise

In developing a new approach to study EE, it is important to investigate the extent to which the approach is reliable, that is to test its repeatability and stability under the different conditions in which it is likely to be used. In this context, it is important to emphasize that variability in measurements of EE encompasses multiple errors that can derive from the instrument, the investigator and the biological variability of the subject, i.e., within-individual variability, day-to-day variability (Donahoo et al., 2004). The contribution of the instrument and investigator error to measures of EE can be minimized by performing (as done in this study), routine maintenance of equipment and the calibration of the calorimeter before each test and using the same measuring equipment for each test: within-instrument errors of <2% and between-machine errors <3% have been reported for EE measured by the Deltatrac (Phang et al., 1990; Wells and Fuller, 1998), the indirect calorimetry system utilized in our studies. As for the contribution of biological variability to the overall error, this was minimized by having a standardized protocol for every subject to follow, all measurements were conducted after overnight fast, and women were all assessed in the follicular phase of their menstrual cycle. Under these conditions, our experiment in nine subjects who repeated the standardized isometric exercise protocol on three separate days reveals a good repeatability for EE at rest and during each exercise level as judged by a mean intra-individual CV of 2.6% for BEE, 4% for EE at each of the five isometric loads applied, and 12% for the slope of the EE-Load relationship, i.e. for the delta energy cost of the isometric exercise. The higher intra-CV obtained for the delta energy cost of the exercise than for EE at each press load (12 vs. 4%) may be explained by the fact that unlike the analysis of each load separately, the regression slope reflects the energy cost through the range of 5 isometric loads (grouped variables). From a mathematical standpoint, the CV of a slope obtained by regression analysis is not comparable to the CV obtained on absolute values: it is more sensitive to small changes in both the numerator and denominator. However, the assessment of the energy cost of the exercise per kg force across a range of loads is a more accurate determination of this parameter than its assessment based upon only 1 or 2 loads.

### EE at rest: measured vs predicted from the EE-load relationship

A consistent finding in the EE-Load relationship is that the y-intercept of this linear regression is significantly higher than for baseline EE at rest (BEE), indicating the metabolic cost of biological processes that remain constant across the various exercise loads. One explanation may be that the EE-Load relationship is not linear, but curvilinear, across the range of very low loads in the range of 0–5 kg force. Alternatively, the higher value of the y-intercept than the BEE value could be reflecting an increased EE associated with the psychobiological conscious “act” of performing the various exercise bouts, increased intra-abdominal pressure, or increased muscle tension in the abdomen/trunk during these types of exercise.

### Application of the standardized test in the postprandial state

This approach developed here for studying isometric thermogenesis can also be applied to investigations in the postprandial state, although there are two prerequisites for this application, namely that the EE-Load relationship should be assessed in a phase of relative steady-state of postprandial EE, and that the time taken to reach this steady-state after the meal ingestion is not too long (say < 1 h). This approach nonetheless underscores the feasibility of its application for investigations into the impact of nutrient composition on the delta energy cost of isometric exercise in the postprandial state.

### Anthropometric correlates and perspective

In line with the main purpose of this study, the criterion to perform intermittent isometric exercise of low intensity with EE comparable with low-level activities of everyday life is met in our study as judged by the findings that EE increases linearly above baseline by 17–62 % on average across the five loads levels (+5 to +25 kg); with the lowest individual value at L1 (+5 kg) and highest individual value at L5 (+25 kg) corresponding to increases in the range of 11–93%. Of particular importance in the methodological development for the assessment of the delta energy cost of isometric exercise is the fact that the inter-CV is several fold greater than the intra-CV, with the slope values ranging from 0.01 to 0.03 (kcal.min^−1^)/kg force t_1/2_. This raises the pertinent question for future research in a large population sample about what could be the determinants of such large inter-individual variability in the energy cost of the standardized isometric exercise (i.e., variability in isometric thermogenesis)?

From an analysis of anthropometric predictors of this large variability in the energy cost of the isometric work, it is shown here (Table 1) that unlike the expected high correlation between baseline EE (at rest) and body weight, there is no significant correlation between the activity EE and body weight expressed to various weight exponents, including those proposed by Prentice et al. (1996) for normalizing various activities for differences in body weight; there was also no correlation with the passive load, i.e., the kg force exerted by positioning the legs on the press-platform but without actively pressing. By contrast, there is a significant correlation with height, suggesting that linear dimensions, but not weight, is a predictor of the large inter-individual variability in isometric thermogenesis in response to the standardized exercise described here. The results of these correlation analyses must, however, be regarded as preliminary and treated with great caution given that it is based on a small sample size (*n* = 9), which while appropriate for methodological validation of feasibility and repeatability, clearly lacks power for providing predictors of inter-individual variability. Large scale studies would be required to confirm the magnitude of the inter-individual variability in isometric thermogenesis, its independency of body weight (and various weight exponents), and to address the importance of stature, the components of stature (lower vs. upper body length), regional body composition and objectively measured fitness in explaining human variability in isometric thermogenesis.

In the meantime, it can be speculated that as this standardized isometric exercise involves specifically the legs in the sitting position, it is most likely that it is leg length rather than height *per se* that explain part of the variability in isometric thermogenesis. Given reports from large epidemiological studies for an association between adult short stature (and/or short leg length) and increased risks for cardiovascular diseases (Paajanen et al., 2010), type 2 diabetes (Asao et al., 2006) and obesity (Hermanussen et al., 2005; Bosy-Westphal et al., 2009), it is tempting to put forward the hypothesis here that low isometric thermogenesis could constitute a metabolic link in the inverse association between stature/leg length and cardiometabolic risks. According to this hypothesis, individuals that have lower stature/leg length would tend to have a lower energy cost for the same workload, and hence spend less energy when performing the same isometric work than individuals with higher stature/leg length. Such energy sparing may constitute a thrifty metabolism link that predisposes people with genetically or environmentally-induced short stature/leg length to increased risks for obesity and cardiovascular diseases.

## Conclusions

This standardized approach to study isometric thermogenesis (in response to intermittent low-intensity isometric workloads), as a complementary approach for EE phenotyping at rest or during dynamic activity, opens up a wide avenue for research in EE and metabolic phenotyping, with implications for research in human energy metabolism, and potential for a better understanding of metabolic predisposition to obesity.

### Conflict of interest statement

The authors declare that the research was conducted in the absence of any commercial or financial relationships that could be construed as a potential conflict of interest.
